# Differential Expression of CXCL12 in Human and Mouse Hair: Androgens Induce CXCL12 in Human Dermal Papilla and Dermal Sheath Cup

**DOI:** 10.3390/ijms26010095

**Published:** 2024-12-26

**Authors:** Mei Zheng, Seungchan An, In Guk Park, Jino Kim, Won-Serk Kim, Minsoo Noh, Jong-Hyuk Sung

**Affiliations:** 1 Epi Biotech Co., Ltd., Incheon 21983, Republic of Korea; mzheng@epibiotech.com; 2 College of Pharmacy, Natural Products Research Institute, Seoul National University, Seoul 08826, Republic of Korea; ann081993@snu.ac.kr (S.A.); ingukpark@snu.ac.kr (I.G.P.); 3 New Hair Institute, Seoul 06134, Republic of Korea; jinokim@inewhair.com; 4 Department of Dermatology, Kangbuk Samsung Hospital, Sungkyunkwan University School of Medicine, Seoul 03181, Republic of Korea; susini@naver.com

**Keywords:** alopecia areata, CXCL12, androgen receptor, dermal papilla, dermal sheath cup

## Abstract

We previously demonstrated that C-X-C Motif Chemokine Ligand 12 (CXCL12) is primarily secreted by dermal fibroblasts in response to androgens and induces hair miniaturization in the mouse androgenic alopecia (AGA) model. However, the direct effects of androgen-induced CXCL12 on dermal papilla cells (DPCs) and dermal sheath cup cells (DSCs) have not been demonstrated. First, we compared single-cell RNA sequencing data between mouse and human skin, and the results show that CXCL12 is highly co-expressed with the androgen receptor (AR) in the DPCs and DSCs of only human hair. Immunohistochemistry also showed that CXCL12 is co-expressed with the AR in the DPCs and DSCs of human hair follicles. In human hair organ culture, androgens also increased CXCL12 expression in DPCs and DSCs and reduced hair length, while the CXCL12 antibody increased hair length via AR inactivation. CXCL12 mRNA was upregulated by androgen treatment in primary human DPCs and DSCs. On the contrary, AR inhibitors or siRNA treatment reduced CXCL12 expression. Collectively, these results suggest that CXCL12 is co-expressed with the AR in the DPCs and DSCs of human hair follicles; therefore, inhibition of CXCL12 using antibodies is a promising strategy for AGA treatment.

## 1. Introduction

C-X-C Motif Chemokine Ligand 12 (CXCL12), a ligand for the G-protein-coupled receptors chemokine (C-X-C motif) receptor 4 (CXCR4) and CXCR7, plays a role in many diverse cellular functions, including embryogenesis, immune surveillance, inflammation response, tissue homeostasis, and tumor growth [1,2]. Recently, it was reported that CXCL12 and CXCR4 are involved in early dermal condensate, but CXCR4 knockout is not required for hair follicle formation [3,4]. Notably, we previously demonstrated that CXCL12 is upregulated in the hair regression period (i.e., catagen and telogen) and induces hair loss via CXCR4 in mice. We also investigated the signaling pathway, finding that treatment with CXCL12 recombinant protein increased the phosphorylation of Stat3 and Stat5, which may play a key role in the process of hair miniaturization [5]. In addition, the CXCL12 level was increased in the dermal papilla region of alopecia areata (AA) patients and is regarded as a stress-sentinel that activates immune cells [6]. We further demonstrated that the CXCL12-neutralizing antibody promotes hair growth in androgenic alopecia (AGA) and AA [7]. In that study, we found that CXCL12 is highly expressed in dermal fibroblasts (DFs) compared with hair follicles in mouse skin. Androgens increase CXCL12 secretion by DFs and upregulate the expression of the androgen receptor (AR) and CXCR4 in dermal papilla cells (DPCs) in a paracrine manner, which is responsible for hair miniaturization in the mouse AGA model. However, the direct effects of androgens on CXCL12 and its expression in DPCs and dermal sheath cup cells (DSCs) have not been reported in AGA because CXCL12 is rarely expressed in mouse DPCs and DSCs [8,9].

AGA is a hair loss condition influenced by genetic and hormonal factors, and the AR plays a key role in AGA progression. CXCL12 and its receptors appear to interact with the androgen signaling pathway; specifically, dihydrotestosterone (DHT) and testosterone upregulate CXCL12 expression through AR activation [7,10,11]. Upon binding of androgens to the AR, the activated receptor translocates to the nucleus, where it interacts with specific DNA sequences known as Androgen Response Elements (AREs), leading to increased CXCL12 and CXCR4 expression [12]. Although the AR is reportedly expressed in the DPCs and DSCs of human hair follicles, the expression of CXCL12 and its functional role in AGA have not been reported, especially in DPCs. In the present study, we examined the expression and functional role of CXCL12 in human hair follicles and further investigated whether it is regulated by the AR in human hair and involved in AGA pathology.

## 2. Results

### 2.1. Expression of Cxcl12 and Ar in Mouse Skin Cells

**Figure 1:**
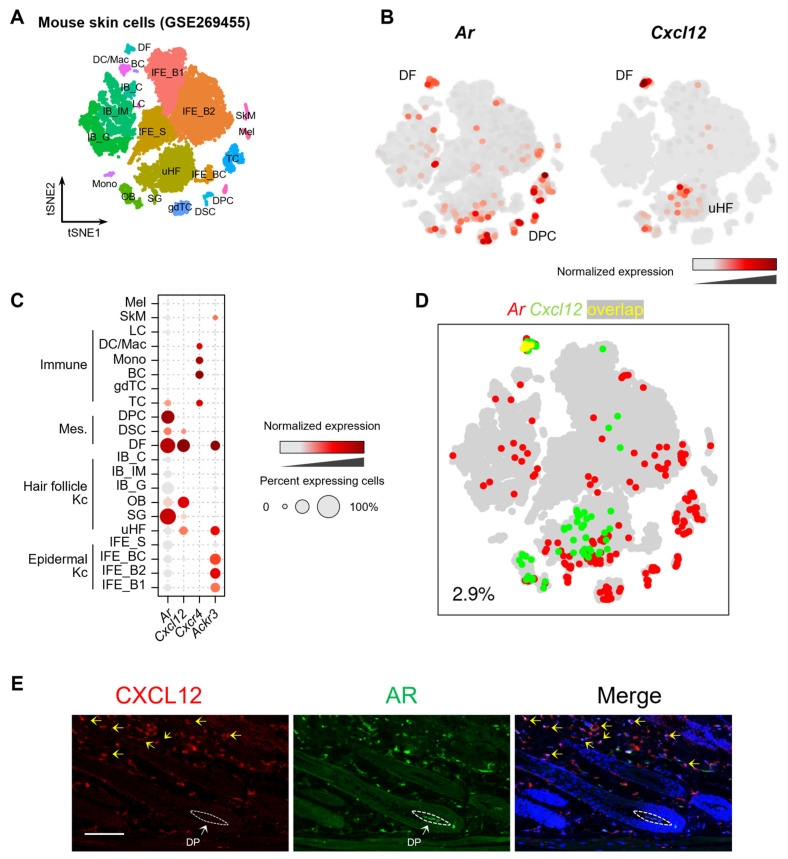
The expression of *Ar* and *Cxcl12* in mouse skin cells. (**A**) t-SNE representation of scRNA-seq results for mouse skin cells, colored by cell type (GSE269455). IFE_B, inter-follicular epidermis (basal); IFE_S, IFE (spinous); uHF, upper hair follicle; SG, sebaceous gland; OB, outer bulge; IB_G, inner bulge (germinative layer cells); IB_IM, IB (medulla); IB_C; IB (cortex); DF, dermal fibroblast; DPC, dermal papilla cell; DSC, dermal sheath cup cell; (γδ)TC, (γδ)T cells; Mono, monocyte; DC/Mac, dendritic cell and macrophage; LC, Langerhans cell; BC, B cell; SkM, skeletal muscle cell; Mel, melanocyte. (**B**) t-SNE representation colored to show the transcript levels of *Ar* and *Cxcl12*. *Ar* is highly expressed in DFs and DPCs. *Cxcl12* is high in DFs but is not abundant in DPCs. (**C**) The dot color and size represent expression levels of *Ar* and *Cxcl12* in skin cells. The color indicates normalized expression levels, and the dot size indicates the percentage of cells expressing the respective gene. (**D**) In the t-SNE representation, cells expressing *Ar* are shown in red, cells expressing *Cxcl12* are shown in green, and cells expressing both are shown in yellow. The percentage (bottom left) represents the proportion of cells expressing both genes among *Ar*-expressing cells. (**E**) Immunohistology of AR and CXCL12 in mouse skin. The AR is highly expressed in the DP; however, CXCL12 is not colocalized in the DP. AR and CXCL12 are colocalized in the DF (indicated by yellow arrows). Scale bar = 100 μm.

The single-cell RNA sequencing (scRNA-seq) analysis of mouse skin (GSE269455) revealed that *Ar* is predominantly expressed in DFs and DPCs (Figure 1A–C). In contrast, *Cxcl12* is highly expressed in DFs but minimally expressed in DPCs (Figure 1B,C). We also searched for information on the expression of *Ar* and *Cxcl12* on the website https://kasperlab.org/mouseskin (accessed on 1 July 2024), which indicated that *Ar* is highly expressed in telogen DPCs, while *Cxcl12* expression is not detected in DPCs (Figure S1). Co-expression analysis further revealed a low percentage of co-expression between *Ar* and *Cxcl12* in mouse skin, particularly in DPCs (Figure 1D). To confirm these transcriptional findings, we examined the protein expression of the AR and CXCL12 in mouse skin tissue using immunostaining (Figure 1E). Consistent with scRNA-seq data, the AR was strongly expressed in DPCs but showed no colocalization with CXCL12 in DPCs, suggesting distinct spatial distributions of these proteins in mouse skin.

### 2.2. Expression of CXCL12 and AR in Human Skin Cells

**Figure 2:**
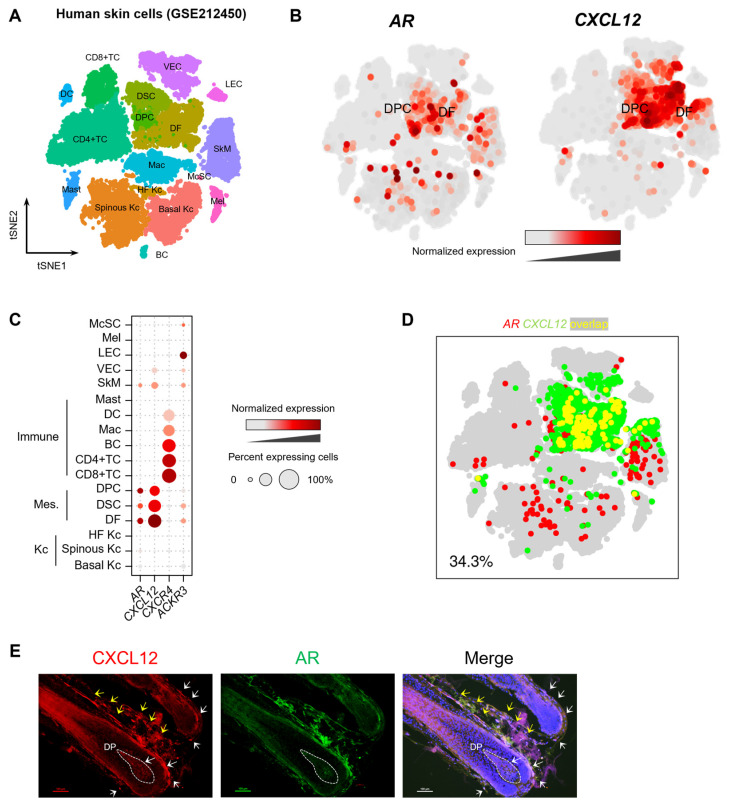
The expression of the *AR* and *CXCL12* in human skin cells. (**A**) t-SNE representation of scRNA-seq results of human skin cells, colored by cell type (GSE212450). Kc, keratinocyte; DF, dermal fibroblast; DSC, dermal sheath cup cell; DPC, dermal papilla cell; TC, T cell; BC, B cell; Mac, macrophage; DC, dendritic cell; Mast, mast cell; SkM, skeletal muscle cell; VEC, vascular endothelial cell; LEC, lymphatic endothelial cell; Mel, melanocyte; McSC, melanocyte stem cell. (**B**) t-SNE representation colored to show the transcript levels of the *AR* and *CXCL12*. The *AR* and *CXCL12* are highly expressed in DFs, DSCs, and DPCs. (**C**) The dot color and size represent the expression levels of the *AR* and *CXCL12* in skin cells. The color indicates normalized expression levels, and the dot size indicates the percentage of cells expressing the respective gene. (**D**) In the t-SNE representation, cells expressing the *AR* are shown in red, cells expressing *CXCL12* are shown in green, and cells expressing both are shown in yellow. The percentage (bottom left) represents the proportion of cells expressing both genes among *AR*-expressing cells. (**E**) Immunohistology of AR and CXCL12 in the human skin. In addition to DFs (yellow arrows), AR and CXCL12 are co-expressed in DPCs and DSCs (white arrows).

The scRNA-seq analysis of human skin (GSE212450) revealed that both the *AR* and *CXCL12* are predominantly expressed in DFs, DPCs, and DSCs (Figure 2A–C). Notably, *CXCL12* exhibited its highest expression in DFs, with moderate expression in DPCs and DSCs. Co-expression analysis further confirmed a high degree of *AR* and *CXCL12* co-expression in human skin, particularly within DFs, DPCs, and DSCs (Figure 2D), suggesting potential functional interactions between these genes in these cell types. To validate these transcriptional findings at the protein level, we performed immunostaining to examine the expression of the AR and CXCL12 in human scalp skin (Figure 2E). Consistent with the scRNA-seq data, CXCL12 was strongly expressed in DFs and moderately expressed in DPCs and DSCs. Importantly, the AR and CXCL12 were found to colocalize within DPCs and DSCs, indicating a spatial relationship between the two proteins in hair follicles.

### 2.3. Hair-Growth-Promoting Effect of CXCL12 Antibody in Testosterone-Induced AGA Models

**Figure 3:**
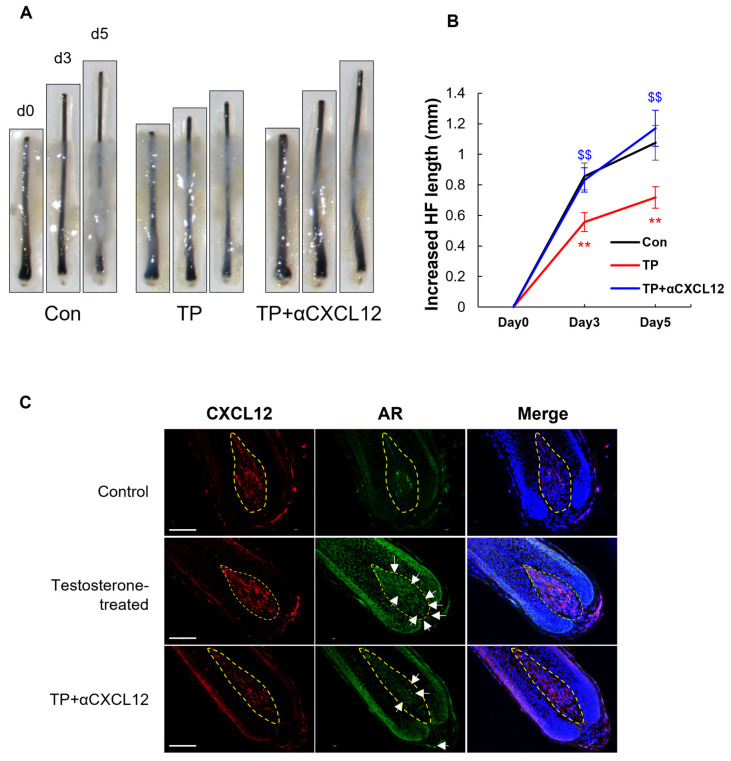
The hair-growth-promoting effect of a CXCL12 monoclonal antibody in a testosterone-induced AGA model. (**A**,**B**) Ex vivo hair organ culture was performed using human hair follicles. Testosterone propionate (TP) decreased hair length, while the CXCL12 monoclonal antibody (αCXCL12) increased hair length on days 3 and 5. ** *p* < 0.01 TP vs. control; $$ *p* < 0.01 TP + aCXCL12 vs. TP (n = 8). (**C**) Immunohistology of the AR and CXCL12 in the human hair follicle on day 5. TP increased AR activation in DPCs (dotted circles: DP), while the CXCL12 monoclonal antibody decreased AR activation (white arrows). Scale bar = 100 μm.

Previously, we demonstrated that the CXCL12 neutralizing antibody (αCXCL12) exhibits a hair-growth-promoting effect in both testosterone propionate (TP)- and DHT-induced AGA mouse models [7]. To further investigate this effect, we evaluated the impact of αCXCL12 on TP-induced AGA using a human scalp hair follicle organ culture model. In this model, treatment with TP significantly inhibited hair growth, as evidenced by a marked reduction in hair follicle length compared to untreated controls (Figure 3A,B). However, the addition of αCXCL12 effectively counteracted the inhibitory effects of TP, resulting in a significant increase in hair follicle length. Similarly, the hair-growth-promoting effect of αCXCL12 was confirmed in the DHT-induced AGA model (Figure S2A,B; see Supporting Information), where αCXCL12 treatment restored hair follicle growth that had been suppressed by DHT exposure.

Confocal imaging provided further insights into the underlying mechanism, revealing increased nuclear translocation of the AR in TP-treated hair follicles. This AR activation was effectively inhibited by the addition of αCXCL12 (Figure 3C), suggesting that αCXCL12 plays a role in modulating androgen-induced AR activation in human hair follicles.

### 2.4. Androgens Increased CXCL12 Expression in DPCs and DSCs Through AR Activation

**Figure 4:**
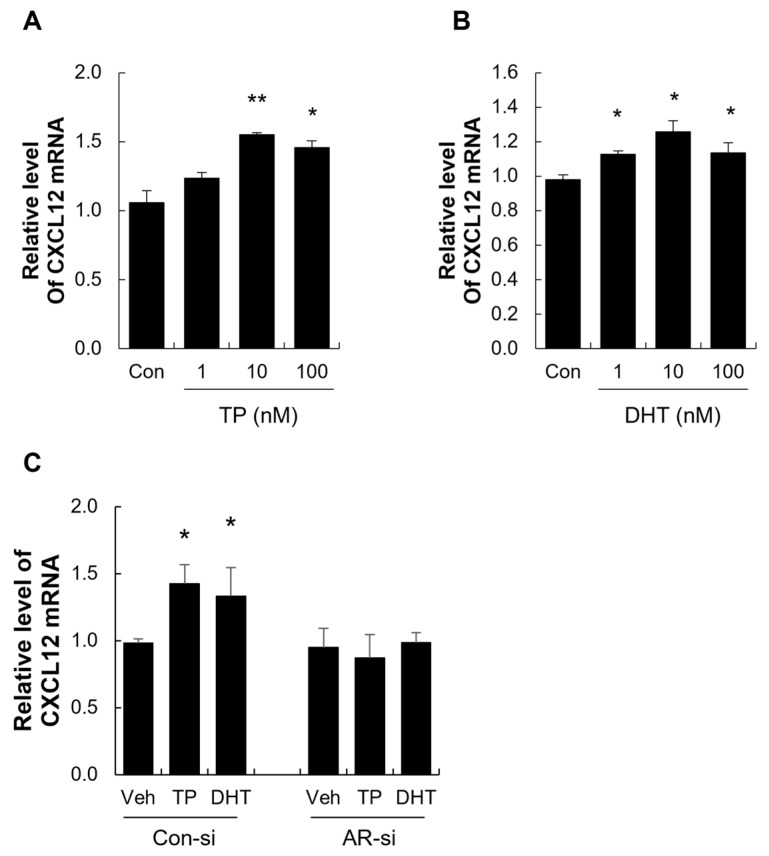
The expression and regulation of CXCL12 in human dermal papilla cells (DPCs). (**A**,**B**) Testosterone propionate (TP, **A**) and dihydrotestosterone (DHT, **B**) increased the CXCL12 level in human DPCs. (**C**) AR knockdown (AR-KD) in DPCs using AR-specific siRNA reduced the expression of CXCL12 induced by TP (100 nM) and DHT (100 nM). * *p* < 0.05; ** *p* < 0.01 vs. control.

**Figure 5:**
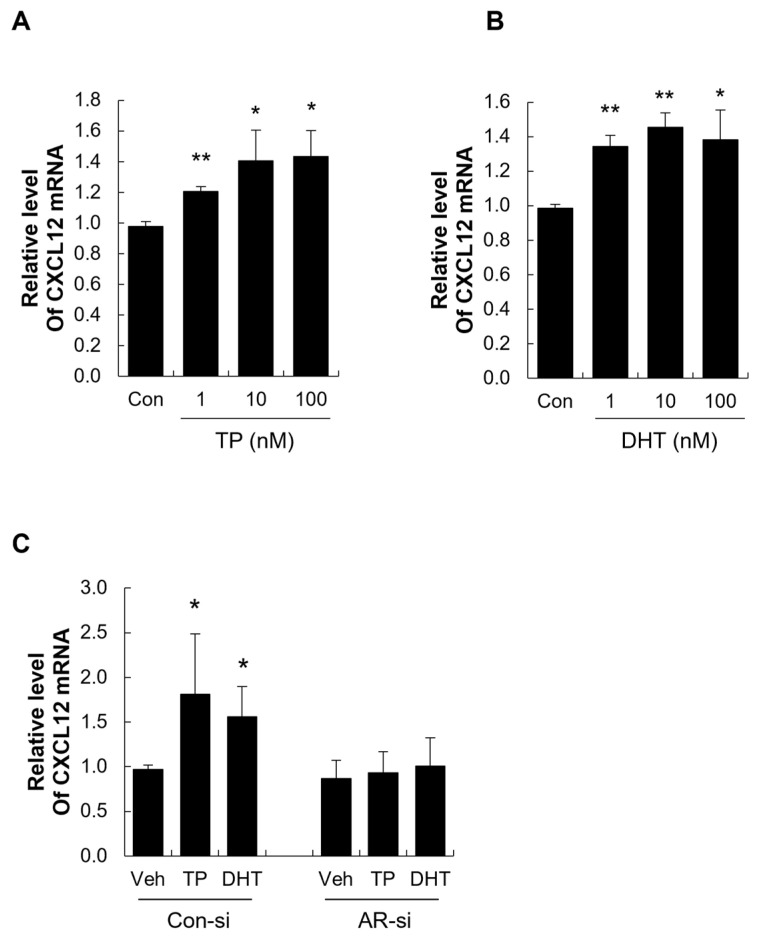
The expression and regulation of CXCL12 in human dermal sheath cup cells (DSCs). (**A**,**B**) Testosterone propionate (TP, **A**) and dihydrotestosterone (DHT, **B**) increased the CXCL12 level in human DPCs. (**C**) AR knockdown (AR-KD) in DSCs using AR-specific siRNA reduced the expression of CXCL12 induced by TP and DHT. * *p* < 0.05; ** *p* < 0.01 vs. control.

To investigate the effect of androgens on CXCL12 expression in human DPCs and DSCs, we treated these cells with varying concentrations of TP or DHT (1–100 nM). Both androgens significantly increased CXCL12 mRNA levels compared to the control (Figure 4A,B and Figure 5A,B).

To determine whether this effect is mediated by the AR, we pre-treated DPCs and DSCs with bicalutamide, the most widely used AR antagonist, before administering DHT or TP. Bicalutamide effectively blocked the androgen-induced upregulation of CXCL12 expression, indicating that the AR is required for this response (Figures S3 and S4). To further confirm the role of the AR in androgen-mediated CXCL12 expression, we performed AR knockdown (AR-KD) in DPCs and DSCs using AR-specific siRNA. AR-KD achieved an approximately 80% reduction in AR mRNA levels in both cell types (Figure S5; see Supplementary Information). Following AR knockdown, the androgen-induced increase in CXCL12 expression was significantly suppressed in both DPCs and DSCs (Figure 4C and Figure 5C). Collectively, these results demonstrate that androgens induce CXCL12 expression in DPCs and DSCs via AR activation, highlighting the critical role of the AR in regulating androgen-mediated hair miniaturization.

## 3. Discussion

In the present study, we examined the expression of CXCL12 in human hair follicles (HFs) and investigated whether it is regulated by the AR in DPCs and DSCs. CXCL12 is co-expressed with the AR in the DPCs and DSCs of only human hair. In isolated human DPCs and DSCs, CXCL12 was upregulated by androgen treatment. On the contrary, AR inhibitors and knockdown reduced CXCL12 expression. In human hair organ culture, androgens also increased CXCL12 expression in DPCs and DSCs and reduced hair length, while the CXCL12 antibody increased hair length. Collectively, these results suggest that CXCL12 is co-expressed with the AR in the DPCs and DSCs of human HFs and plays a key role in AGA progression.

**Figure 6:**
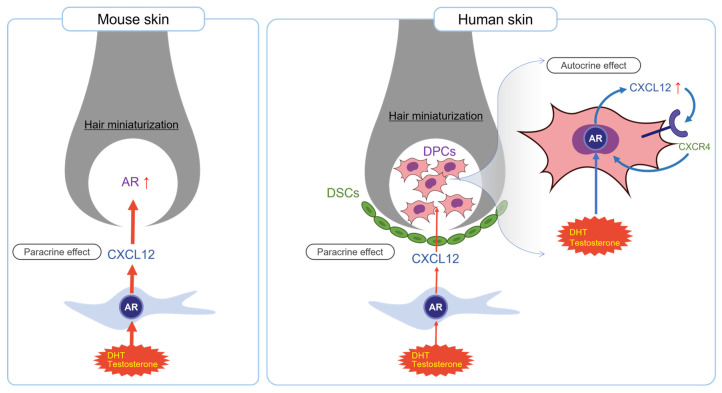
A potential mechanism of CXCL12-mediated hair miniaturization in AGA. In mouse skin, CXCL12 is primarily expressed in dermal fibroblasts and acts in a paracrine manner. In human skin, CXCL12 is co-expressed with the AR in DPCs and DSCs, exerting both paracrine and autocrine effects via CXCR4 and the AR.

As described above, after its upregulation by androgens, CXCL12 influences HF maintenance and contributes to the hair miniaturization observed in AGA. In a previous study, we reported the specific localization of CXCL12 in mouse skin, where it is highly expressed in DFs, in addition to ORS cells [8,13]. The CXCL12 secreted by these cells within the HF microenvironment interacts with its receptor CXCR4, which is highly expressed in immune cells and DPCs [8]. In mouse skin, CXCL12/CXCR4 signaling activated by androgens (via paracrine effects) ultimately causes HFs to transition from the anagen (growth) phase to the catagen/telogen phase of the hair cycle, disrupting the normal hair growth process [5]. However, in human skin, CXCL12 is highly expressed and colocalizes with the AR in DPCs and DSCs in addition to DFs. CXCL12 secreted by DFs can induce hair miniaturization in a paracrine manner. Notably, androgens such as DHT and testosterone increased the expression of CXCL12 in DPCs and DSCs via AR activation, and the secreted CXCL12 itself could induce hair miniaturization in an autocrine and paracrine manner (Figure 6).

The AR is a pivotal transcription factor primarily associated with AGA. The AR was highly expressed in the mesenchymal cells and weakly detected or not detected in the epithelial components of hair follicles. The AR is reportedly expressed in DPCs and testosterone, and DHT inhibited hair regrowth by activating the AR in DPCs [14]. For example, the nuclear localization of the AR was significantly increased in the DPCs of AGA-affected balding scalps, and the AR induced the regression of blood vessels in the DP to mediate hair miniaturization [15]. DPCs from the balding scalps of AGA patients underwent premature senescence and had high AR expression [16]. In the present study, we first demonstrated a different hair loss mechanism, where androgens primarily increased the CXCL12 levels in DPCs and DSCs, which increased the expression of the AR in an autocrine manner in DPCs and DSCs to induce hair miniaturization.

In addition to AR activation and hair miniaturization, immune cell infiltration has been reported in AGA hair analysis [17]. For example, single-cell analysis (GSE36169) revealed that γδT cells, activated CD8+ T cells, effector memory CD4+ T cells, eosinophils, and neutrophils were significantly increased in the bald scalp [18]. Michel et al. also performed a microarray analysis, and inflammatory cytokines such as CXCL12 and CCL18 were found to be increased in AGA-affected scalps [19]. It is reasonable to assume that androgens and the AR induce moderate to severe inflammatory responses in the scalps of AGA patients [19,20]. Although we did not further investigate the involvement of the CXCL12/CXCR4 pathway in the immune cell infiltration of AGA hair follicles, single-cell analysis in alopecia areata revealed that CXCR4 is highly expressed on immune cells, such as CD4+ T cells, CD8+ T cells, and dendritic cells, and is co-expressed with inflammatory cytokines and chemokines [8]. It is reasonable to assume that CXCL12 is an inflammation-related gene, and its increased expression in AGA can promote an inflammatory response in the scalp by acting as a chemoattractant, which may lead to immune cell infiltration. This inflammatory response and immune cell infiltration can alter the microenvironment around the hair follicles, negatively affecting hair growth in AGA. Therefore, we should pay attention to the regulation of the expression of CXCL12 in order to mitigate the progression of AGA.

Hair loss results from various factors, and current treatments for AGA include topical medications (minoxidil) and oral drugs (5-α reductase inhibitors). Since 5-α reductase inhibitors are administered daily, developing a long-lasting solution is crucial for enhancing the effectiveness and convenience of AGA treatments. Notably, CXCL12 antibody therapy has many advantages in AGA treatment. Because the molecular weight of the antibody is very high (>150 kDa), it is slowly absorbed through the lymphatic system [21,22]. Additionally, the direct injection of antibody medications into hair-loss areas is possible, leading to superior efficacy without systemic toxicity. Therefore, the inhibition of CXCL12 using humanized antibodies is a promising approach for AGA treatment.

## 4. Materials and Methods

### 4.1. Meta-Analysis of scRNA-Seq Data

We performed a meta-analysis of publicly available single-cell RNA sequencing (scRNA-seq) data from mouse and human studies. The dataset for mouse skin cells was obtained from the Gene Expression Omnibus (GEO) under accession number GSE269455 [8], and the human dataset was retrieved from GSE212450 [23]. Both datasets were processed using the Seurat v5.0.3 R package for quality control and cell clustering, as previously described [8,24,25].

For both datasets, low-quality cells were filtered out based on the following criteria: cells with more than 10% mitochondrial gene expression or fewer than 200 detected genes. The remaining high-quality cells were clustered based on gene expression patterns, and cell populations were annotated using well-established marker genes.

In the mouse dataset (GSE269455), basal interfollicular epidermis (IFE_B) cells were annotated using Krt5 and Krt14 as markers, while suprabasal IFE (IFE_S) cells were identified by the expression of Krt1 and Krt10. Cycling basal IFE (IFE_BC) cells were marked by Krt5, Krt14, Stmn1, and Mki67. Upper hair follicle (uHF) cells were characterized by Krt17 and Krt79, while sebaceous gland (SG) cells were annotated using Scd1 and Mgst1. Outer bulge (OB) cells were identified by the expression of Barx. Hair follicle keratinocytes were categorized based on Krt27 and Krt35 expression and further subdivided into the germinative layer (IB_G), inner root sheath and medulla (IB_IM), and cortex/cuticle (IB_C) cells, depending on the expression of proliferative markers like Stmn1. Fibroblast-like cells were differentiated into dermal fibroblasts (DFs) based on Col1a1 and Lum expression, and dermal papilla cells (DPCs) were identified by the expression of Corin and Notum. Dermal sheath cells (DSCs) were classified based on Ramp1 and Mylk expression. Immune cells were classified according to their specific marker genes, with T cells (TCs) identified by Cd3e, gamma-delta T cells (γδTCs) by Trdc, monocytes (Mono) by Cd14 and Ccl6, dendritic cells and macrophages (DCs/Mac) by Cd68 and Cd74, Langerhans cells (LCs) by Cd207, and B cells (BCs) by Cd79a. Additionally, skeletal muscle (SkM) cells were identified by Acta1 and Des, while melanocytes (Mel) were characterized by the expression of Pmel and Dct.

In the human dataset (GSE212450), keratinocytes (KCs) were identified by the expression of KRT5 and KRT14, while hair follicle keratinocytes (HF KCs) were marked by KRT10 and SOX9. DFs were annotated based on COL1A1 expression. DSCs were identified by COL11A1, and DPCs were characterized by CORIN and WNT5A. TCs were classified using the marker CD3D, while BCs were annotated by the expression of CD79A. Macrophages (Mac) were identified by CD86 expression, dendritic cells (DCs) by CLEC9A, mast cells by HPGD, SkM cells by TAGLN, and endothelial cells (ECs) by PECAM1. Melanocytes (Mel) were marked by MITF, and melanocyte stem cells (McSCs) were distinguished by the expression of SOX10.

### 4.2. Hair Organ Culture

During organ culture, hair growth activity of human HFs was observed. Adult scalp HFs were obtained from the upper lip region using a scalpel and forceps. Isolated HFs were placed in a defined medium (Williams E medium supplemented with 2 mM L-glutamine, 10 µg/mL insulin, 10 ng/mL hydrocortisone, 100 U/mL penicillin, and 100 μg/mL streptomycin, without serum). Individual vibrissa HFs were photographed 3–5 days after the start of the incubation. Changes in hair length were calculated from the photographs and expressed as the mean ± standard error (SE) of 8 vibrissae HFs.

### 4.3. Immunostaining

For immunofluorescence studies of human hair follicles, 5 μM formalin-fixed and paraffin sections were used. After heating, antigen retrieval skin sections were stained with primary antibodies, including anti-CXCL12 (R&D systems, Minneapolis, MN, USA) and anti-AR (Santa Cruze Biotechnology, Dallas, TX, USA). Alexa Fluor 488 or Alexa Fluor 594-conjugated goat anti-rabbit or goat anti-mouse antibody was used as the secondary antibody (Invitrogen, Carlsbad, CA, USA). Immunofluorescence images were captured using a Nikon Eclipse Ts2 microscope (Nikon, Tokyo, Japan).

### 4.4. Cell Isolation and Cell Culture

Individual HFs were supplied by the New Hair Institute (Seoul, Republic of Korea). All biopsies were performed with full patient written consent. Additionally, the Ethical and Scientific Committees of the participating institution confirmed the present study to be in accordance with the ethical standards (as laid out in the 1964 Declaration of Helsinki). Human hair follicle dermal papilla and dermal sheath cup were microdissected from the HFs under a stereomicroscope and cultured. hDPCs and hDSCs were cultured in CellCor™ DPC CD (Xcell therapeutics, Seoul, Republic of Korea) with 1% antibiotic–antimycotic (Thermo Fisher Scientific, Waltham, MA, USA) and maintained in a humidified incubator at 37 °C with 5% CO_2_.

### 4.5. RNA Extraction, cDNA Synthesis, and Quantitative Real-Time PCR (qRT-PCR)

Quantitative real-time PCR (qRT-PCR) reactions were conducted using the StepOne Real-Time PCR System (Applied Biosystems, Foster City, CA, USA). Total cellular RNA was extracted using Invitrogen TRIzol Reagent (Thermo Fisher Scientific) and then subjected to reverse transcription using a cDNA synthesis kit (Nanohelix, Daejeon, Republic of Korea). The primer sequences used for the experiments were as follows (forward and reverse), respectively: 5′-ATTCTCAACACTCCAAACTGTGC-3′ and 5′-ACTTTAGCTTCGGGTCAATGC′ for *CXCL12*; 5′-CGAAGACGACAAGATGGACAA-3′ for *AR*, 5′-CATCACTGCCACCCAGAAGACTG-3′ and 5′-ATGCCAGTGAGCTTCCCGTTCAG-3′ for *GAPDH*.

### 4.6. Knockdown of AR In Vitro

Transfections were conducted using Lipofectamine RNAiMAX reagent (Invitrogen) following the manufacturer’s protocol. To silence endogenous AR expression, a pool of small interfering RNAs (siRNAs), consisting of a mixture of three siRNAs provided as a single reagent, was used. The sequences of the siRNAs are as follows: si-AR-#1 sense, 5′-AUACUUGAAGGGUAGAUUC-3′ and anti-sense, 5′-GAAUCUACCCUUCAAGUAU-3′; si-AR-#2 sense, 5′-UAGAGAGACAGGGUAGACG-3′ and anti-sense, 5′-CGUCUACCCUGUCUCUCUA-3′; si-AR-#3 sense, 5′-UCUUCGGCUGUGAAGAGAG-3′ and anti-sense, 5′-CUCUCUUCACAGCCGAAGA-3′.

### 4.7. Statistical Analysis

All data are demonstrated as the means ± standard deviations of at least three independent experiments. For analysis between two groups, Student’s *t*-test was used. When more than two groups were compared, a one-way analysis of variance (ANOVA) test followed by Tukey’s post hoc test was used. The significance values were set as follows: * or $ *p* < 0.05, ** or $$ *p* < 0.01 and *** *p* < 0.001. GraphPad Prism 5.01 (GraphPad Software Inc., San Diego, CA, USA) was used for all statistical analyses.

## Data Availability

The data presented in this study are available in the Gene Expression Omnibus (GEO) at https://www.ncbi.nlm.nih.gov/geo/query/acc.cgi?acc=GSE269455 for the mouse dataset (GSE269455) and https://www.ncbi.nlm.nih.gov/geo/query/acc.cgi?acc=GSE212450 for the human dataset (GSE212450), both accessed on 1 July 2024.

## Supplementary Materials

The supporting information can be downloaded at: https://www.mdpi.com/article/10.3390/ijms26010095/s1.
