# Supplementary material for: Differential Expression of CXCL12 in Human and Mouse Hair: Androgens Induce CXCL12 in Human Dermal Papilla and Dermal Sheath Cup
Source: Int J Mol Sci. 2024 Dec 26;26(1):95. doi: 10.3390/ijms26010095 (PMC11719931; doi:10.3390/ijms26010095)
Supplement: Supplementary file 1 [file ijms-26-00095-s001.zip › ijms-3370778-supplementary.pdf]

Different expression of CXCL12 in human and mouse hair: androgens induce CXCL12 in human dermal papilla and dermal sheath cup

Mei Zheng[1,#], Sungchan An[2,#], In Guk Park[2,#], Jino Kim[3], Won-Serk Kim[4], Minsoo Noh[2,*] and Jong-Hyuk Sung[1,*]

[1]Epi Biotech Co., Ltd. Incheon 21983, South Korea

[2]College of Pharmacy, Natural Products Research Institute, Seoul National University, Seoul, 08826, Republic of Korea

[3]New Hair Institute, Seoul 06134, South Korea

[4]National University, Department of Dermatology, Kangbuk Samsung Hospital, Sungkyunkwan University School of Medicine, Seoul, 03181, Republic of Korea

*Co-correspondence:

College of Pharmacy, Natural Products Research Institute, Seoul National University, Seoul, 08826, Republic of Korea. minsoonoh@snu.ac.kr (M. Noh, Ph.D.);

Epi Biotech Co., Ltd., Incheon 21983, Republic of Korea. brian99@epibiotech.com (JH Sung, Ph.D.)

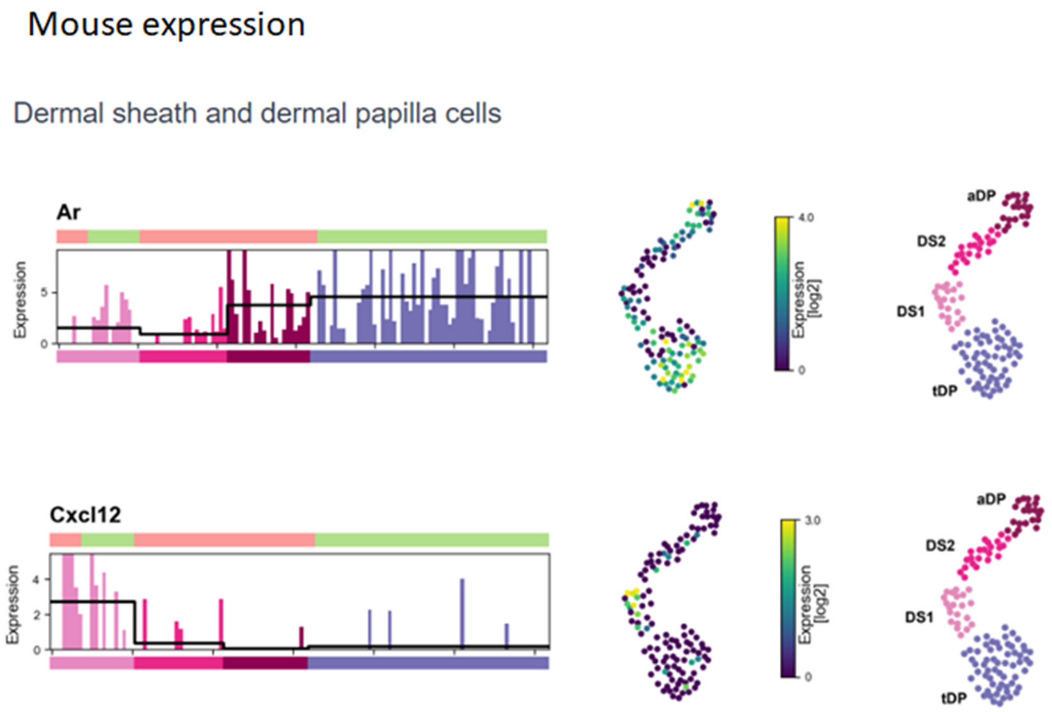

Figure S1. Expression of *Ar* and *Cxcl12* in mouse DPCs and DSCs. scRNA-seq results of mouse skin cells (GSE129218). *Ar* is expressed in DPCs and DSCs, upregulated in telogen DPCs. *Cxcl12* is expressed in DSCs.

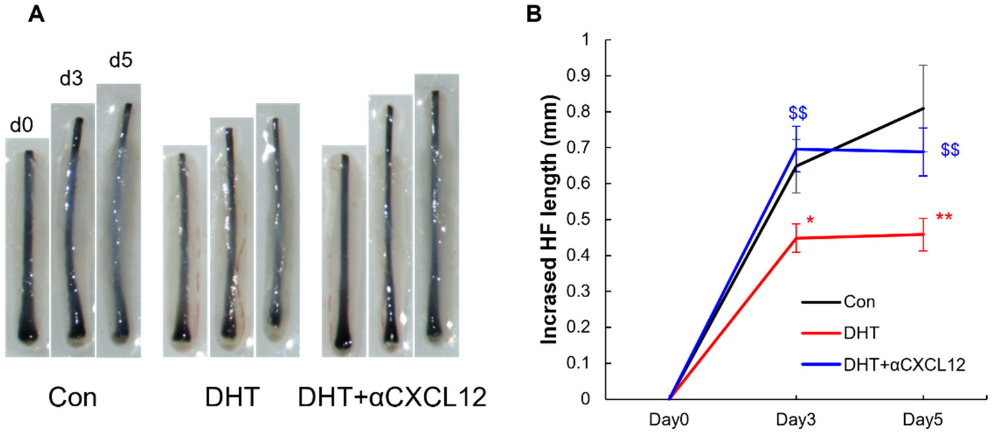

Figure S2. Hair-growth promoting effect of CXCL12 monoclonal antibody in testosterone-induced AGA model. (A, B) Ex vivo hair organ culture was performed using human hair follicle. DHT decreased the hair length, while CXCL12 monoclonal antibody increased the hair length at day 3 and day 5. **: DHT vs Control, $$: DHT+aCXCL12 vs DHT.

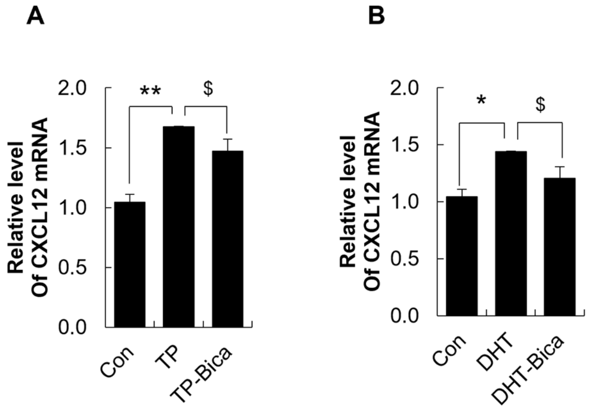

Figure S3. The effect of AR antagonist in androgens-induced CXCL12 expression in DPCs. Bicalutamide (500nM) pre-treatment inhibited 100nM of TP (A) and 100nM of DHT (B)-induced upregulation of CXCL12 expression. *, **: vs control, $: vs TP or DHT.

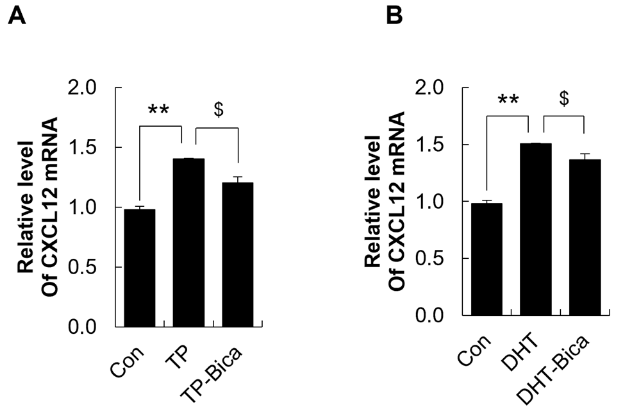

Figure S4. The effect of AR antagonist in androgens-induced CXCL12 expression in DSCs. Bicalutamide (500nM) pre-treatment inhibited 100nM of TP (A) and 100nM of DHT (B)-induced upregulation of CXCL12 expression. **: vs control, $: vs TP or DHT.

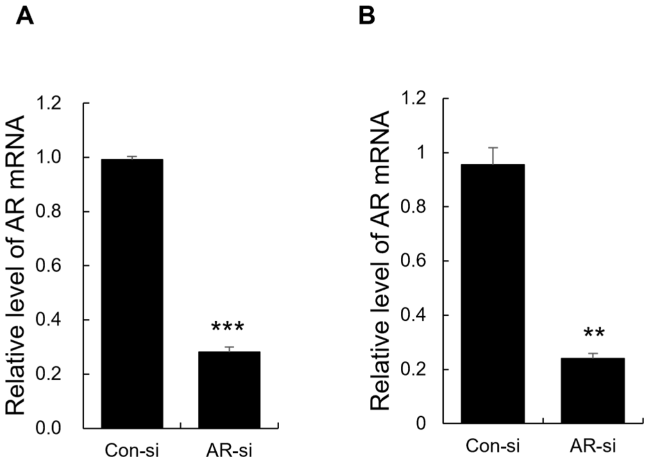

Figure S5. Knockdown of AR in DPCs and DSCs. Endogenous AR expression was silenced by transfecting cells with a pool of siRNA targeting AR, and the knockdown efficiency was confirmed by qRT-PCR in DPCs (A) and DSCs (B). ** and ***: vs control.
